# Tumor-targeted costimulation by using bi-specific aptamers

**DOI:** 10.14800/ccm.1333

**Published:** 2016-06-06

**Authors:** Fernando Pastor

**Affiliations:** 1 Instituto de Investigación Sanitaria de Navarra (IDISNA), Recinto de Complejo Hospitalario de Navarra, Pamplona 31008, Spain; 2 Program of Molecular Therapies, Aptamer Unit, Centro de Investigación Medica Aplicada (CIMA), Pamplona, 31008, Spain

**Keywords:** Aptamer, Costimulation, Cancer Immunotherapy

## Abstract

Aptamers are chemically synthesized oligonucleotides that can be easily engineered for cancer immunotherapy use. So far, most of the therapeutic aptamers described are antagonistic and block the function of a receptor or its soluble ligand. Recently, aptamers have been modified to act as agonists by multimerization, with a direct application in cancer immunotherapy. Several agonistic aptamers against costimulatory receptors have been described. However, systemic costimulation, though potentially a very potent antitumor immune strategy, is not devoid of auto-inflammatory side effects. In a quest to reduce toxicity and improve efficacy – reducing the therapeutic index – the first bi-specific aptamers to target the costimulatory ligand to the tumor have been described, showing very promising results in different preclinical tumor models.

Cancer immunotherapy has become a reality upon the recent success of clinical trials in very aggressive tumors with immune-checkpoint blockade antibodies [1, 2]. These strategies are aimed at modulating the antitumor immune response. The FDA approval of ipilimumab (anti-CTLA4 monoclonal antibody) or ipilimumab and pembrolizumab (anti PD1 monoclonal antibodies) has been a milestone in cancer immunotherapy [1, 2]. The great results obtained in these trials have precipitated the initiation of a large amount of new clinical trials with immunomodulatory agents in the last few years. However, the antitumor immune response can be tackled not only by immune-checkpoint blockade, but also by favoring T-cell activation, providing artificial costimulatory signals to the tumor-reactive lymphocytes [3]. That has been initially achieved by using agonistic antibodies to costimulatory receptors, such as 4-1BB, OX40, GIRT, etc [4]. Antibodies are bivalent molecules that facilitate the dimerization of the cognate receptor triggering the activation by crosslinking. In the last few years several agonistic antibodies have reached the clinical trial pipeline based on the promising results obtained in animal models [3]. Still, these approaches have an important caveat that needs to be overcome: the high associated toxicity. It has been broadly documented in pre-clinical and clinical settings that this sort of therapy is usually associated with toxicity. Reducing the threshold of T-cell activation would also facilitate the initiation of auto-inflammatory responses that could be very deleterious in some cases [5]. 4-1BB agonistic antibodies elicited severe autoimmune hepatitis in preclinical and clinical trials, with high lymphocytic hepatic infiltration [6, 7]. And CD28 superagonist antibodies have elicited lethal cytokine storms in phase-I clinical trials [8]. Besides, the field is leading towards the combination of different immunomodulatory agents that would act in synergism, eliciting a more powerful antitumor immune response [9]. The addition of each new immunomodulatory agent to the therapeutic combo would not only enhance the antitumor immune response, but it would also exacerbate the auto-inflammatory immune responses. Considering the need to reduce the side effects associated with these cancer immunotherapy strategies, a reasonable approach to improve the therapeutic index would be to deliver the costimulatory artificial ligand only to the tumor, favoring the activation of tumor-reactive lymphocytes and precluding the activation of non-desirable autoreactive immune responses in other parts of the body. Initially that has been achieved by using chimeric recombinant proteins with dual specificities; one arm of the protein binds to a tumor membrane receptor, and the other one elicits T-cell activation, as it happens with the BiTEs. An example of this approach is a recombinant antibody that interacts with CD19 and CD3 Blinatumomab simultaneously eliciting T-cell activation in the vicinity of B cells recently FDA approved for leukemia treatment [10]. These sorts of approaches have shown very potent antitumor immune responses [11]. The downside is that the chimeric recombinant protein could be immunogenic, eliciting neutralizing antibodies that would preclude its use in consecutive treatments. The linker region of the chimera protein could be recognized by the immune system as a foreign antigen, increasing the chances of triggering a neutralizing immune response. For Blinatumomab it might not be much of a problem as it depletes B lymphocytes precluding the induction of neutralizing antibodies, but for other type of tumor targeting receptors it needs to be considered.

Aptamers, which are single-stranded oligonucleotides that have been selected throughout a complex interactive process named SELEX (Systematic Evolution of Ligands by Exponential Enrichment), are artificial ligands that display specificities and affinities similar or superior to monoclonal antibodies [12]. Aptamers have also been named chemical antibodies, because they are not cell-based products. Indeed, they are chemically synthesized compounds, and that has a direct impact on good manufacture production, reducing cost and complexity in the regulation process. Aptamers cannot only be easily modified to adjust their pharmacokinetics, but they can also be conjugated with other cargoes of different nature through straightforward chemical reactions to deliver them to specific target cells [13]. Here I will focus on how to deliver another aptamer to the tumor by engineering bi-specific aptamers. Several years ago, while I was working in Gilboa’s team, we managed to generate the first bi-specific aptamer to deliver costimulation to tumor cells [14]. Few years before, the same group developed the first-in-class agonistic aptamer against 4-1BB [15], which was achieved by dimerization of the artificial 4-1BB ligand aptamer. Since then, other agonistic aptamers to different costimulatory receptors have been described (OX40, CD28, CD40), all of them following the same principle: to trigger the crosslink of the receptor to deliver the activation signal [16–18]. The costimulation induced by these agonistic aptamers is similar or even in some cases superior to the corresponding agonistic antibody. In order to facilitate the accumulation of the agonist costimulatory aptamer only in the tumor, we link the agonistic dimeric aptamer with a tumor-specific aptamer. The first bi-specific aptamer bound to PSMA and 4-1BB receptor was able to trigger 4-1BB costimulation in PSMA-expressing tumor and not in the parental ones. It was able to elicit a potent antitumor immune response at much lower doses than the non-targeting 4-1BB agonists. That was translated into a reduction of the auto-inflammatory toxicity associated with the systemic 4-1BB stimulus [14]. The improvement in mice survival was correlated with a high infiltration of lymphocytes in the tumor. The bi-specific aptamer was tested in melanoma and colon carcinoma models artificially modified to express a non-internalizing PSMA. In order to display a therapeutic effect, the targeted tumor receptor cannot be internalized, which accounts for the most common type of receptors. Targeting 4-1BB costimulation to the tumor was compared with the gold standard vaccine Gvax; the bi-specific aptamer was proved to elicit a more robust antitumor response than the Gvax vaccine. In a further study by Schrand *et al* [19], they developed a bi-specific aptamer to deliver 4-1BB costimulation to the tumor stroma by targeting the soluble protein VEGF which is expressed in a large number of tumors and therefore has a broad application. This is a very clever approach that allows killing two birds with one stone, blocking the proangiogenic factor VEGF while at the same time providing a potent costimulatory signal to activate only the tumor infiltrating lymphocytes through 4-1BB agonistic aptamer. Although it could be presumed that the lack of 4-1BB immobilization might render a weaker immune response, it turns out to be a very potent approach in several different types of tumors –spontaneous and orthotopic tumor models of melanoma, fibrosarcoma, breast cancer and glioblastoma [19]. The VEGF-41BB bi-specific aptamer has shown a better therapeutic index than the non-targeting 4-1BB costimulatory agonist (including the monoclonal antibody). Also, in the quest to generate a bi-specific aptamer that could be used to target different types of tumors, we engineered an MRP1-CD28 bi-specific aptamer, binding to MRP1 expressed in a broad amount of tumors with chemotherapy resistance and in cancer stem cells [20]. MRP1 is a marker that accounts for the most aggressive tumors, resistant to conventional chemotherapy drugs and highly metastatic [21, 22]. The MRP1-CD28 aptamer was able to increase tumor infiltration in a melanoma model enriched in cancer stem cells. The treatment with MRP1-CD28 bi-specific aptamer potentiates a vaccine-induced immune response, reducing tumor growth and improving mice survival [20].

The bi-specific aptamers could also be used to generate an ex-vivo whole-cell cancer vaccine [20]. The quest for cell-specific cancer vaccines has been dramatically pursued in cancer immunotherapy [23]. It has been proved in preclinical models that a tumor-cell vaccine genetically modified to express immune-stimulatory cytokines or receptors is able to elicit a potent antitumor immune response with very significant antitumor effects [24]. Nevertheless, the translation of this approach to the clinic is technically cumbersome, as it implies generating a genetically modified stable cell line derived from each tumor patients and, in most cases, it is not easy to derivate a primary tumor-cell line.

A future perspective, considering that tumors are adapting constantly to positive selection pressures such as chemotherapy or the antitumor immune response, implies that it would be desirable to tackle the tumor from two different flanks to avoid its progression. The tumor has great plasticity and can adapt to the vast majority of the current treatments in a longer or shorter period of time. Therefore, the idea is to foresee how the tumor would adapt and try to redirect the tumor evolution down a route of your own interest. For instance, in order to overcome chemotherapy, the tumor upregulates MRP1, which would open a window for cancer immunotherapy intervention by targeting costimulation to MRP1. Then, the selection pressure would be on MRP1-expressing cells pushing the tumor to evolve towards low MRP1-expression variants, again sensitive to chemotherapy. Thus, alternating these two types of treatments would make it possible to contain the tumor progression for a much longer time.

## Figures and Tables

**Figure 1:**
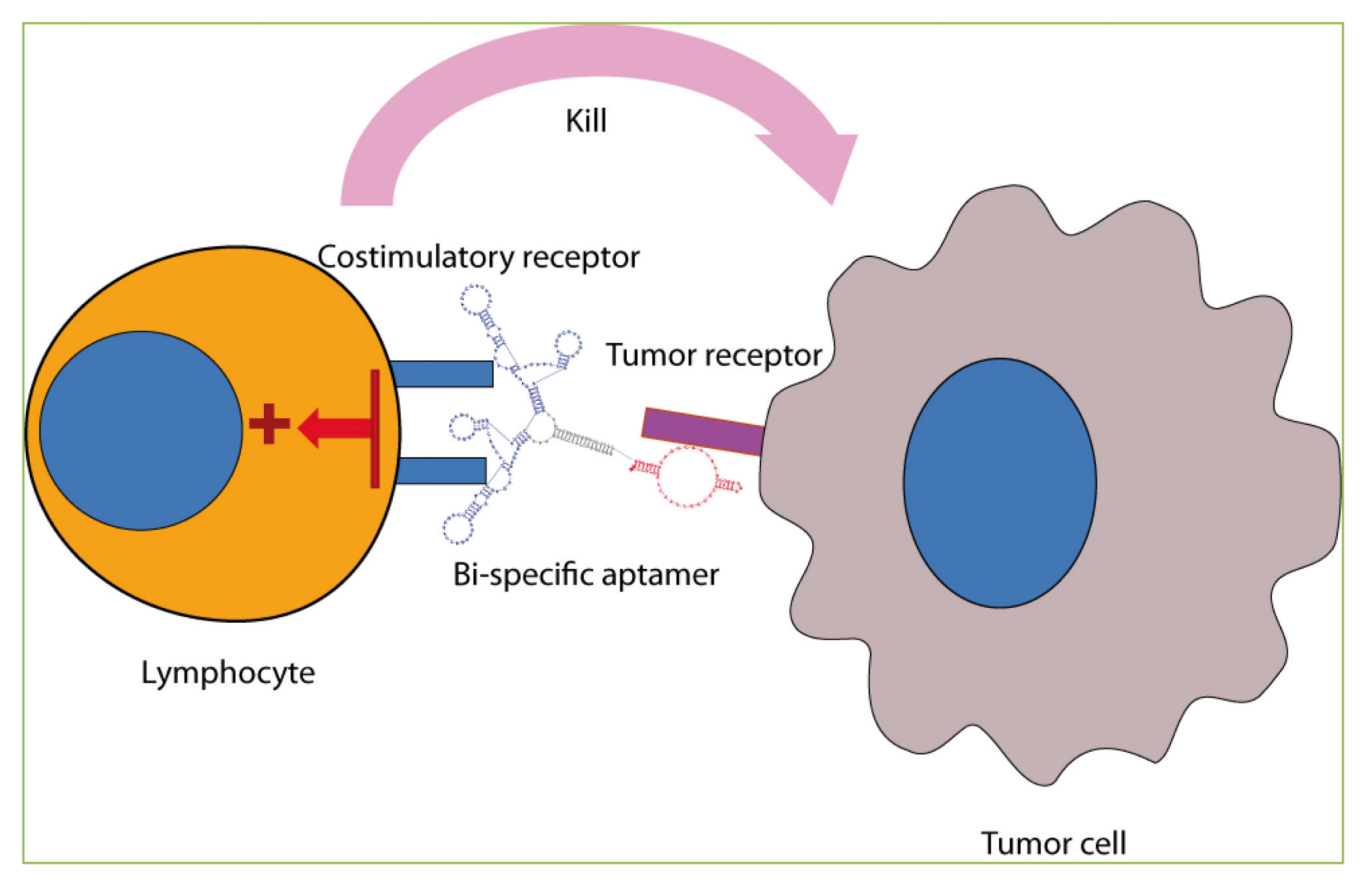
Bi-specific tumor-targeting costimulatory aptamers facilitate the activation of T lymphocytes in the proximity of the tumor-target cell.
